# Nursing Care for Stroke Patients: Current Practice and Future Needs

**DOI:** 10.3390/nursrep13030106

**Published:** 2023-09-10

**Authors:** Lisa A. Babkair, Razan A. Safhi, Raghad Balshram, Rahaf Safhei, Atheer Almahamdy, Fatimah Hamad Hakami, Ali Matouq Alsaleh

**Affiliations:** 1 Faculty of Nursing, King AbdulAziz University, Jeddah 21589, Saudi Arabia; rsafhi0002@stu.kau.edu.sa (R.A.S.); rblsharm@stu.kau.edu.sa (R.B.); rsafhi0001@stu.kau.edu.sa (R.S.); aalmahamdy0001@stu.kau.edu.sa (A.A.); 2 King Fahad General Hospital, Jeddah 23325, Saudi Arabia; fatimahhh@moh.gov.sa; 3 National Neuroscience Institute Nursing Administration, King Fahad Medical City, Riyadh 12231, Saudi Arabia; amalsaleh@kfmc.med.sa

**Keywords:** critical care, nursing training, nursing practice, stroke, stroke patients, stroke unit

## Abstract

Background: Stroke is the second leading cause of death and the third leading cause of disability worldwide. Stroke nurses play an important role in the care of patients living with stroke by using best practices and adhering to stroke-management guidelines. This study aims at examining the current nursing practice for stroke patients in Saudi Arabia. Method: A cross-sectional descriptive design was used to collect data from nurses working in the stroke unit and intensive care unit between the period of February and June 2022 using electronic self-administered questionnaires. Results: A convenience sample of 131 nurses who provided care for stroke patients was enrolled. Significant differences in nursing practice were found between the stroke units and the intensive care units regarding the activation of the stroke code, *X*^2^ (4, *N* = 131) = 48.34, *p* < 0.001; transferring stroke patients to a designated bed, *X*^2^ (4, *N* = 131) = 48.74, *p* = 0.002; applying the NIHSS, *X*^2^ (4, *N* = 131) = 70.11, *p* < 0.001; using the modified Rankin scale, *X*^2^ (4, *N* = 131) = 61.24, *p* < 0.001; providing intervention for neglect syndrome, *X*^2^ (4, *N* = 131) = 44.72, and hemianopsia, *X*^2^ (4, *N* = 131) = 39.22; screening for poststroke depression, *X*^2^ (4, *N* = 131) = 101.59, *p* < 0.001; assessing for psychosocial needs, *X*^2^ (4, *N* = 131) = 74.44, *p* < 0.001, and encouraging patients to express their feelings, *X*^2^ (4, *N* = 131) = 58.64, *p* < 0.001; educating patients and families about stroke prevention, *X*^2^ (4, *N* = 131) = 40.51, *p* < 0.001. Conclusion: As per the results of the study, there is an urgent need for stroke units run by specialized stroke nurses to provide early stroke management and improve survivors’ outcomes. Structured stroke-care programs are needed to improve nursing practice and meet the international standard of stroke care.

## 1. Introduction

Stroke is the second leading cause of death and the third leading cause of disability worldwide. Every year, 15 million individuals worldwide suffer from a stroke, with one-third dying and another one-third of survivors living with a permanent disability [1].

Incidences of stroke in Saudi Arabia have been increasing in recent decades because of its aging population. A recent systematic review reported that incidences of stroke in Saudi Arabia are 29 per 100,000 people annually [2]. Stroke is a devastating disease that affects stroke survivors’ quality of life, causes severe physical disability, and increases mortality [3].

In Saudi Arabia, the Ministry of Health (MOH) is the major body supporting the medical services. Currently, Saudi Arabia has over 350 hospitals with a limited number of stroke centers [4]. In fact, just 5% of stroke patients in Saudi Arabia are admitted to acute stroke units and receive complete stroke care from specialists [5]. As a result, the present stroke services and staff numbers are insufficient to provide care to the increased number of expected stroke-incidence patients.

Because of the paucity of stroke centers in several Saudi Arabian regions, most stroke patients are commonly treated in the intensive care unit (ICU). Nurses in the ICU are currently providing care for stroke patients. Moreover, stroke nurses in Saudi Arabia generally gain their qualifications by the number of years of working experience with stroke patients.

Stroke nurses play a significant role in the care of stroke patients through appropriate practices, starting from following stroke-management guidelines in providing advanced care to stroke patients. Additionally, they focus on the patient’s comprehensive care from the point the patient is admitted into the hospital until the patient is discharged to a rehabilitation department. Furthermore, stroke nurses provide a plan of care to ensure continuity of care for stroke patients after discharge. Therefore, implementing a stroke-care system will ensure advanced stroke management and provision of a high quality of care during acute and recovery care.

The focus in the acute phase appears to be on the “time is brain” principle. The principle emphasizes the time-sensitive necessity of early detection and management in patients with suspected strokes [6]. It is important that nurses be able to recognize stroke manifestations early and determine the onset of signs and symptoms to ensure that patients can receive appropriate treatment within a time window [7].

Furthermore, the use of several stroke scales can also be beneficial to detect stroke manifestations and assess the severity of a stroke [8]. Several studies have suggested that the National Institutes of Health Stroke Scale (NIHSS) is effective, and it is widely used, easy to learn, and rapidly completed on admission. It is considered the gold standard for determining the severity of a stroke. Based on the scale scores, nurses can determine the need for advanced stroke care for stroke patients [9].

According to previous studies on the early management of patients with stroke, one of the important members of the stroke-management team is the nursing staff [8]. Stroke nurses are well prepared to provide urgent care for stroke patients. According to the American Heart Association (AHA) and the European Consensus Group, patients with stroke need immediate medical attention. Early diagnosis of stroke and rapid hospitalization, primarily thrombolytic therapy, (tPA) tissue plasminogen activator and early rehabilitation, and early secondary prevention will reduce stroke mortality [10]. The purpose of all these practices is to address the diagnosis of the condition, achieve medical stability, and prevent early complications in the acute period of the stroke. Starting medical and nursing interventions as early as possible for stroke patients will enhance patient outcomes and minimize stroke complications.

Furthermore, nursing plays a vital role in the care of poststroke after the first 72 h of care [11] Poststroke nursing care focuses on rehabilitation services and secondary prevention measures to enhance stroke survivors’ outcomes and avoid stroke recurrence. A study reported that appropriate nursing care and the provision of support from patients’ families influences effective rehabilitation for stroke survivors [10]. Stroke has an impact on the patient’s physical and psychological well-being, as well as their social and occupational integration [12]. The nurse’s role is to follow up with the patient during the recovery phase to ensure continuity of care. However, stroke survivors might face care interruption during the transition from hospitals to rehabilitation or home. Therefore, nursing care practice should also focus on following up with stroke survivors to reduce the risk of readmission and improve the quality of survivors’ lives [13].

Even today, the psychosocial health of a patient with a stroke remains under-recognized and undertreated [14]. According to the AHA and the American Stroke Association’s (ASA) scientific statement, depression following a stroke is a dynamic phenomenon that affects up to one-third of stroke survivors [15]. Poststroke depression (PSD) among stroke survivors is associated with decreased functional independence and high stress levels, poor cognitive recovery, longer time to resume social activities, lower quality of life, and higher mortality rates. Nursing care guidelines are effective in guiding nursing practice to enhance the social integration of stroke survivors and screen survivors for PSD [16,17,18,19].

Furthermore, a study reported that years of experience of working with stroke patients improved nursing practice. A number of years of experience of acute stroke care increases stroke awareness and improves the actual practices associated with care [20]. In fact, obtaining a specialized certification, such as a stroke nursing certification, ensures a high quality of advanced care for patients with strokes. In some countries, working in specialized neuro-units requires that nurses must be certified to be competent to work in specialized clinical areas. However, as mentioned earlier, most of the stroke nurses in Saudi Arabia obtain their title from previous clinical experience with stroke patients.

A stroke is a medical emergency that needs immediate medical attention. Therefore, specialized stroke services, such as stroke units and specialized healthcare providers, will ensure that advanced stroke management is conducted during the periods of hyperacute, acute, and postacute care [21]. The continued lack of understanding of current nursing practices for the care of stroke patients has resulted in a lack of knowledge of the healthcare development needs, negatively affecting patient outcomes and quality of care. In Saudi Arabia, the number of stroke units is limited. Few studies have investigated nursing care for stroke patients in Saudi Arabia. This study aims at examining the current nursing practice for stroke patients in Saudi Arabia.

## 2. Materials and Methods

### 2.1. Research Design

This study employed a quantitative, descriptive, cross-sectional design to collect data about current nursing practice for stroke patients.

### 2.2. Setting and Sample

The study was conducted at three MOH hospitals: King Fahad Medical City (KFGH) is a nonprofit organization with a total capacity of 1200 beds, and it is one of the largest and fastest-growing medical complexes in the Middle East; King Fahad General Hospital (KFGH) in Jeddah is considered as one of the biggest MOH hospitals in the western region hospitals, with a 628-bed capacity; King Abdullah Medical Complex (KAMC) in Jeddah is a 500-bed hospital. The reason for selecting these hospitals was either that they had specialized stroke centers or were eligible to provide treatment for strokes.

A convenience sample of approximately 131 nurses was selected from the three hospitals. This study included head nurses, in-charge nurses, and registered nurses who provided direct care to stroke patients in the stroke unit or intensive care unit (ICU). New nursing staff in training or nursing interns were excluded from the study.

### 2.3. Recruitment and Data Collection

The participants were recruited after the researcher obtained the ethical permission for the study using the following procedure. The principal investigator sent an email to each hospital’s nursing supervisor, along with a letter of invitation, an ethical approval form, and a document containing the study information. Then, the nursing supervisors sent the information to the heads of nursing at the selected units, including the link for the SurveyMonkey questionnaire. Finally, the heads of nursing emailed the questionnaire to the nursing staff.

The data in this study were collected using two questionnaires. The sociodemographic questionnaire consisted of eight items: nurses’ age, gender, degree of education, working position, years of experience as a nurse, years of experience working with stroke patients, working sites, and working unit.

The second questionnaire focused on nursing practices with stroke patients and was composed of 71 items: prehospital care (7 items), acute care (30 items), and postacute care (34 items). The participants’ responses were measured by using a 5-point Likert scale, as follows: (1) strongly disagree, (2) disagree, (3) neutral, (4) agree, and (5) strongly agree. The researchers followed the following steps in developing the nursing practices for stroke patient questionnaire. The researchers developed the questionnaire after reviewing the literature and considering the most important practices in dealing with stroke patients according to the guidelines published by the AHA/ASA, European guidelines, and Saudi MOH guidelines. First, the researchers made the aim of the study a focal point of the questionnaire. Second, they selected a 5-point Likert scale to ascertain the degree of participant agreement with each nursing practice. Third, they determined the main dimensions of the questionnaire based on the available guidelines for stroke care. Last, each practice was selected carefully and placed under its associated dimension. All practices related to stroke-patient care were selected based on the authors’ review of the literature and current stroke-care guidelines [19,22,23]. After completing the first draft of the questionnaire, the researchers submitted the questionnaire to an expert panel, which consisted of four nursing experts working in a stroke unit who had several years of experience in caring for stroke patients. The researchers revised the questionnaire three times according to the feedback from the expert panel. The approved version of the questionnaire consisted of three dimensions: prehospital care, acute care, and postacute care. The internal consistency of the questionnaire was calculated and found to be highly reliable. The prehospital-care subscale consisted of 7 items (α = 0.84), the acute-care subscale consisted of 30 items (α = 0.88), and the postacute-care subscale consisted of 34 items (α = 0.87).

### 2.4. Data Analysis

Data were analyzed via SPSS version 26, with a descriptive analysis in the form of means, percentages, frequencies, and standard deviations. Further, an independent chi-square test was conducted to examine the differences in nursing practices between stroke units and the ICUs.

## 3. Results

### 3.1. Sociodemographic Characteristics

A sample of 131 nurses completed an online questionnaire. Table 1 shows the demographic characteristics of the sample. The nurses were relatively young (M = 33.27, SD = 5.82) and the ages ranged from 23 to 51 years. The majority of the sample (107; 81.7%) were females. The majority of nurses (107; 81.7%) had a bachelor’s degree in nursing, whereas 105 (80.2%) nurses were staff nurses. Nearly half of the sample (47; 35.9%) had nursing experience of more than 10 years, and 64 (48.9%) nurses had five years of nursing experience with stroke patients. There were 52 (39.7%) nurses from KFMC, 35 (26.7%) from KFGH, and 44 (33.6%) from KAMC. More than half of the nurses, 73 (55.7%), were working in the ICU and 58 (44.3%) nurses were working in stroke units.

### 3.2. Survey of Nursing Practices for Stroke Patients

Table 2 presents the average score for nurses’ responses to each nursing care practice for patients with stroke. Regarding the first dimension, prehospital care, the majority of the nurses agreed that nursing practices with stroke patients during the triaging phase, and nurses’ ability to recognize associated medical histories, clinical signs and symptoms, and risk factors for stroke, are common practice. However, some participants were neutral about the activation of the stroke code practice (*M* = 3.18, *SD* = 1.70) and the rapid transfer of a stroke patient to a designated bed (*M* = 3.23, *SD* = 1.66).

In the second dimension, acute care, the majority of the nurses agreed that most nursing practices with stroke patients during this phase were applicable. However, some nurses disagreed or were neutral about the need to assess stroke severity by applying the NIHSS (*M* = 3.19, *SD* = 1.76), obtaining NIHSS 24 h post-tPA administration (*M* = 3.21, *SD* = 1.69), obtaining NIHSS scores before conducting a procedure (*M* = 3.18, *SD* = 1.65), and obtaining NIHSS scores after conducting a procedure (*M* = 3.30, *SD* = 1.63).

Regarding the third dimension, postacute care, the majority of the nurses agreed that most nursing practices with stroke patients during this phase are applicable. However, some nurses disagreed or were neutral regarding nursing practices for the assessment of physical disability (*M* = 3.41, *SD* = 1.65), providing intervention for neglect syndrome (*M* = 3.50, *SD* = 1.50) and hemianopsia (*M* = 3.64, *SD* = 1.46), screening for PSD (*M* = 2.76, *SD* = 1.72), the assessment of psychosocial needs (*M* = 3.09, *SD* = 1.69), the encouragement of patient-feeling expression (*M* = 3.39, *SD* = 1.56), and educating patients and families about stroke prevention (*M* = 3.75, *SD* = 1.50).

### 3.3. Nursing Practices for Stroke Patients across Units

Further statistical analysis was conducted to determine whether there was a significant difference in nursing practices for stroke patients between the stroke units and the ICUs. We performed a chi-squared test of independence to examine the differences in each nursing practice and the two clinical units, stroke unit, and ICU. Table 3 shows the significant differences between nursing practices across units during prehospital care, including the activation of the stroke code, *X*^2^ (4, *N* = 131) = 48.34, *p* < 0.001, and transferring stroke patients to designated beds, X^2^ (4, *N* = 131) = 48.74, *p* < 0.001. Both nursing practices were more applicable in the stroke unit than in the ICU.

During the acute phase, we also found several significant differences in nursing practices across the two units. Table 4 shows that assessing stroke severity by applying the NIHSS was significant, *X*^2^ (4, *N* = 131) = 70.11, *p* < 0.001; many nurses agreed that this scale was more commonly used by nurses in the stroke unit than in the ICU. Moreover, evaluating the inclusion and exclusion eligibility criteria for tPA administration was also significant, *X*^2^ (4, *N* = 131) = 20.14, *p* < 0.001, between the two units. Furthermore, a similar significant difference was found with obtaining the NIHSS score 24 h post-tPA administration, *X*^2^ (4, *N* = 131) = 85.90, *p* < 0.001, obtaining the NIHSS before the procedure, *X*^2^ (4, *N* = 131) = 84.61, *p* < 0.001, and obtaining the NIHSS after the procedure, *X*^2^ (4, *N* = 131) = 78.08, *p* < 0.001.

During the postacute phase, we detected some major differences in nursing practice across two units. Table 5 shows that assessing patient physical disability using the modified Rankin scale (mRS) or other tools was significant, *X*^2^ (4, *N* = 131) = 61.24, *p* < 0.001. Many nurses reported that this scale was used more frequently in the stroke unit than in the ICU. Moreover, providing an intervention for neglect syndrome was significant, *X*^2^ (4, *N* = 131) = 44.72, *p* < 0.001. This practice was more prevalent in the stroke unit than the ICU. We also found similarly significant values with providing intervention for hemianopsia, *X*^2^ (4, *N* = 131) = 39.22, *p* < 0.001. There was a significant difference in screening for poststroke depression, *X*^2^ (4, *N* = 131) = 101.59, *p* < 0.001. Assessment of psychosocial needs, *X*^2^ (4, *N* = 131) = 74.44, *p* < 0.001, and the encouragement of patients to express their feelings was also significant, *X*^2^ (4, *N* = 131) = 58.64, *p* < 0.001, between the two units. Furthermore, we found a significant difference in providing education to patients and families about stroke prevention and encouraging follow-up clinic after discharge, *X*^2^ (4, *N* = 131) = 40.51, *p* < 0.001 between two units.

## 4. Discussion

This study examined current nursing practices for stroke patients in Saudi Arabia across three dimensions: pre-, acute, and poststroke care. Regarding the first dimension, prehospital care, our results showed that most nurses recognize stroke signs and symptoms. Consistent with previous studies, the identification and consistent use of a standardized assessment was found to provide reliable and consistent data. A possible explanation for this similarity is that the FAST test is easy to use and is a highly recommended screening tool when a patient presents with the signs and symptoms of a stroke [24].

Regarding prehospital care, the data revealed that 40% and 39%, respectively, of nurses disagreed with the application of activating the stroke code and the rapid transfer to designated bed practices. According to the AHA and ASA guidelines, the increased time taken from symptom onset to admission to an emergency department (ED) is the major cause of ineligibility for acute reperfusion treatments. This could be due to the lack of patients, and the public understanding of stroke signs and symptoms and of the necessity of immediate care. However, Saudi Arabia has provided a unified emergency number, as with 911, that might be used to create a faster medical and public response. The delay in the activation of the stroke code might be due to uncertainty with stroke onset and symptoms.

Regarding the second dimension, acute care, the data indicated that 40.5% of nurses disagreed about using the NIHSS in the ICU. Our study showed that most of those who did use it, 42%, were stroke-unit nurses. In line with another study, it was found that the use of the NIHSS is limited outside the stroke unit [25]. One explanation for this could be that nurses in other units, such as ICU nurses, are unfamiliar with this scale, or they do not use it frequently; therefore, nurses might need to be trained to use the NIHSS [25].

A total of 41% of ICU nurses reported they did not perform the NIHSS 24 h post-tPA administration, while 52% agreed to perform this practice. Nurses should frequently assess patients with the NIHSS after receiving tPA treatment to identify if there is a neurological deterioration [26]. Another study found that using the NIHSS can help manage and evaluate therapeutic effectiveness post-tPA administration and identify early complications [27]. It is well known that the patient’s neurologic status changes within the first 24 h following intravenous thrombolysis therapy [28]. It is possible that this variation in NIHSS use is due to the fact that our nurses still need more training sessions on using stroke-tools assessment.

The analysis confirmed that the evaluation of inclusion and exclusion eligibility criteria for tPA administration was performed more in the stroke unit than in the ICU. This result is consistent with earlier findings. Treatments, including intravenous tPA, were used more frequently at primary stroke centers [29]. Moreover, stroke patients who were treated in stroke centers were more likely to receive tPA [30]. Although the stroke unit in Saudi Arabia is still being developed and improved, these findings are likely obtained because stroke nurses are more knowledgeable about stroke care than other nonspecialized nurses.

The current study found that, in stroke units, 42% of nurses reported applying the mRS or other measures to assess patient physical disability more frequently than in the ICU. Comparing the results with those of another study, they found that the mRS is the most widely used major outcome measure for acute stroke. Additionally, nurses use other assessment tools with the mRS to address the defects and provide a more comprehensive understanding of poststroke disability [31]. Healthcare agencies must grasp the importance of involving nurses in rehabilitation services. They should clarify the roles of nurses in stroke rehabilitation and develop systematic procedures to ensure adequate time is provided for rehabilitation nursing care. In fact, the mRS is widely used as a measure of long-term functional outcomes in community settings after a stroke [31].

There may be potential complications after a stroke according to the AHA scientific statement (2021). Nurses and other healthcare professionals should prevent and manage these complications. Neglect syndrome is one of the major complications associated with a stroke [32]. According to this study’s findings, stroke nurses are willing to deal with neglect syndrome. Further, this practice is applied more in the stroke unit than in the ICU. This finding is consistent with a qualitative study that found most nurses adopt various behaviors to increase patient awareness of their left side. This includes verbally telling patients to pay attention to their left side or picking up something from their left side [33]. Moreover, it was mentioned in a prior study that nurses play a key role in hemianopsia management, as well as in educating the patients and their families [34]. The reason behind this might be that approximately half of the nurses in this study, 48.9%, had at least five years of experience working with stroke patients.

According to our survey, a significant percent, 52%, of participants disagreed about using the poststroke depression (PSD) screening. This finding is supported by a recent study conducted in Saudi Arabia. The study reported that the majority of stroke survivors are discharged from hospitals without receiving PSD screening [14]. Additionally, although nurses recognize the necessity of screening and detecting depression symptoms, they rarely utilize recommended depression-screening tools [19]. Furthermore, incidences of PSD increase when stroke survivors lack social support and experience social isolation. We found similar results regarding the lack of psychosocial support, which further validated our findings from the perspective of a lack of attention to psychosocial needs. As evidenced by previous studies, stroke survivors with PSD have reported a lack of perceived social support, restricted social involvement, and a poor quality of life [14]. In line with our findings, there is a lack of practices encouraging patients to express their feelings. A possible explanation for this could be that most of the practices are focused on acute-stage concerns, whereas postacute care, including consideration of the patient’s psychosocial status, is mostly ignored. The literature has reported that healthcare providers pay little attention to the psychological state of stroke patients [14]. According to another study, clinical care practices for stroke patients are mainly focused on general care, regardless of the stage of the disease. This could be because the nurses focus on stroke priority outcomes rather than rehabilitation nursing care [35]. Furthermore, generally, most hospitals are lacking transition-of-care programs from the stroke unit to home care, in which patients get lost in the middle of the care [36].

Patient education after a stroke is an important element to prevent stroke recurrence. Family members require education sessions to learn skills to provide home care for stroke survivors. However, in this study, about 28% of the ICU nurses disagreed about patient-education practices in the ICU. The AHA/ASA highly recommend post-stroke patient education to prevent stroke recurrence [22]. One explanation for the study’s finding is that stroke patients are usually admitted to the ICU during the acute stage and are discharged once they are medically stable, which means that the ICU nurse would not be able to provide education to the patient and their family members during the acute stage.

There are some limitations to consider in this study in using convenience sampling, which increases the risk for selection bias. In Saudi Arabia, there are no equivalent national studies on nursing practices with stroke patients. Moreover, the number of stroke-care units available is limited and has fewer nurses to participate in the study than the ICU. Therefore, the heterogeneity of the results of some nursing practice between the ICU and stroke unit was clearly identified in the analysis of the study. However, this is the first study in Saudi Arabia to examine evidence-based nursing practice in stroke care. The researchers compared stroke units to the ICUs in three hospitals, in which the gap in nursing practice across clinical areas should receive further attention to improve quality of stroke care. Further, the survey for this study was developed based on standardized stroke-care guidelines.

Based on the study findings, we recommend the development of a stroke unit for patient care in several Saudi Arabian regions. This will help in early stroke identification and allow advanced stroke management within the treatment window. It will also help improve the application of evidence-based practices and standardized guidelines in providing appropriate interventions for patients with strokes. Moreover, we recommend providing educational and training programs to critical nurses to increase their knowledge and skills to use the NIHSS. During the rehabilitation phase, we recommend nurses provide and pay adequate support and attention to stroke survivors. Psychosocial support plays an important role in stroke survivors’ and their families’ health. Based on current poststroke recommendations, early depression screening is one of the most critical screenings among stroke survivors. Hospitals should have a clear policy for screening stroke patients for depression. Nurses should be trained on using depression-screening tools for early-depression-symptom identification to improve patient outcomes. Further research is needed to examine how nurses can improve stroke outcomes in different care units, regardless of whether they are stroke units or ICUs. Moreover, stroke care should be provided with specialized competent stroke nurses. Further examination for obtaining stroke certification from an accredited body is highly recommended for nurses to work in specialized stroke areas. This will improve clinical outcomes for stroke patients and prevent long-term complications in stroke survivors.

## 5. Conclusions

In this study, we aimed to examine current nursing practices and determine the future nursing needs of patients with strokes in Saudi Arabia. We found a significant variation between several nursing practices across stroke units and ICUs regarding the activation of the stroke code, transferring stroke patients to a designated bed, applying the NIHSS, using the modified Rankin scale, providing intervention for neglect syndrome and hemianopsia, screening for poststroke depression, assessing for psychosocial needs and encouraging patients to express their feelings, and educating patients and families about stroke prevention. These practices were more prevalent in the stroke unit than in the ICU. However, the majority of nurses agreed that most care practices for stroke patients were applicable. There is a variability of standards in some aspects of nursing stroke care, and, as a result, there is scope for their improvement. A stroke-care program and specialized certification is highly recommended for nurses who provide care for patients with strokes.

## Figures and Tables

**Table 1:** Sociodemographic characteristics of the sample.

Characteristics		(N)	(%)
Gender	Male	24	18.3
	Female	107	81.7
Educational degree	Diploma	18	13.7
	Baccalaureate	107	81.7
	Master’s	6	4.6
Current clinical position	Head nurse	7	5.3
	Charge nurse	19	14.5
	Staff nurse	105	80.2
Years of experience as a nurse	Less than one year	7	5.3
	1–5 years	41	31.3
	6–10 years	36	27.5
	More than 10 years	47	35.9
Years of experience with stroke patients	Less than one year	11	8.4
	1–5 years	64	48.9
	6–10 years	37	28.2
	More than 10 years	19	14.5
Working hospital	Hospital at Riyadh	52	39.7
	Hospital-1 at Jeddah	35	26.7
	Hospital-2 at Jeddah	44	33.6
Working unit	Stroke unit	58	44.3
	Intensive care unit	73	55.7

**Table 2:** Nursing care practice for stroke patients.

Practices	Mean	Std. Deviation
**Dimension 1: Prehospital care**		
**1.1.** **Triaging phase**		
1.1.1. Identify stroke signs and symptoms (FAST)	4.21	1.162
1.1.2. Recognize the onset of stroke symptoms from the last time patient was seen as normal	4.10	1.073
1.1.3. Activate stroke code	3.18 *	1.703
1.1.4. Initiate rapid transfer to a designated bed	3.23 *	1.667
**1.2.** **Recognize medical history and lifestyle risk factors for onset of stroke, including**		
1.2.1. Medical/surgical history	4.14	1.080
1.2.2. Medication/anticoagulant history	4.21	1.065
1.2.3. Stroke risk factors	4.19	1.039
**Dimension 2: Acute care**		
**2.1.** **Acute care**		
2.1.1. Prepare for CT/CTA scan as soon as possible	4.55	0.825
2.1.2. Assess airway, breathing, and circulation	4.51	0.863
2.1.3. Connect patient on cardiac monitor/monitor vital signs	4.56	0.860
2.1.4. Assess stroke severity by applying the National Institutes of Health Stroke Scale (NIHSS)	3.19 *	1.763
2.1.5. Evaluate inclusion and exclusion eligibility criteria for tPA administration	4.07	1.242
2.1.6. Monitor blood glucose and provide treatment if <60 mg/dL according to physician’s orders	4.43	0.912
2.1.7. Monitor and manage blood pressure appropriately and in accordance with physician’s orders	4.52	0.862
2.1.8. Recognize targeted blood pressure needed for specific patient with stroke	4.48	0.906
2.1.9. Identify and differentiate stroke signs/symptoms and stroke mimics	4.44	0.870
2.1.10. Obtain 12 leads ECG and identify abnormal cardiac rhythm	4.43	0.895
2.1.11. Maintain oxygen saturation > 94%	4.44	0.896
2.1.12. Establish intravenous access, two eighteen-gauge cannula if possible	4.48	0.817
2.1.13. Administer appropriate fluids according to stroke type and following physician’s orders	4.47	0.816
2.1.14. Assess need for NG/OG tube-insertion and catheterization-insertion before tPA administration	4.41	0.944
2.1.15. Perform initial bedside swallowing screening	4.25	1.018
2.1.16. Assess for transient ischemic attack (TIA) risk and recurrence using a proper tool	4.31	0.961
**2.2.** **Post-tPA administration and considerations**		
2.2.1. Monitor vital signs post-tPA	4.47	0.853
2.2.2. Transfer to the hyperacute stroke unit or ICU if needed, and attach to a cardiac monitor for 72 h post-tPA as per protocol	4.45	0.787
2.2.3. Perform neurological assessment post-tPA	4.53	0.835
2.2.4. Apply precautions post-tPA	4.48	0.862
2.2.5. Monitor for thrombolytic complications	4.52	0.798
2.2.6. Position patient appropriately post-tPA	4.46	0.834
2.2.7. Obtain the NIHSS score 24 h post-tPA administration	3.21 *	1.699
**2.3.** **Pre- and postoperative management**		
2.3.1. Obtain the NIHSS score before the procedure	3.18 *	1.659
2.3.2. Conduct preprocedural assessment, including vital signs, medical history, allergies, medications, and laboratory results	4.50	0.817
2.3.3. Discuss sedation with physician and document the blood-pressure plan	4.43	0.804
2.3.4. Monitor vital signs and conduct neurological assessment at least every 30–60 min preprocedure	4.51	0.768
2.3.5. Obtain the postprocedural NIHSS score	3.30 *	1.639
2.3.6. Check vital signs and conduct neurological assessment, check procedure site, and conduct postprocedural-circulation assessment	4.48	0.788
2.3.7. Monitor for postprocedural complications	4.53	0.778
**Dimension 3: Postacute care**		
3.1. Provide oxygen therapy and suctioning as needed	4.59	0.773
3.2. Monitor for signs of deterioration	4.59	0.793
3.3. Monitor for deep-vein-thrombosis (DVT) signs	4.58	0.784
3.4. Identify previous falls and assess risk for falls	4.60	0.751
3.5. Monitor for signs of infection	4.55	0.796
3.6. Conduct oral-hygiene assessment and oral care as needed	4.57	0.775
3.7. Manage nasogastric feeding tube safely	4.59	0.773
3.8. Assess for risk of aspiration	4.63	0.778
3.9. Assess nutritional need and nutritional deficiency	4.56	0.776
3.10. Assess for bowel constipation and urine retention and manage it by following physician’s orders	4.60	0.752
3.11. Assess patient physical disability using the modified Rankin scale or other tools	3.41 *	1.650
3.12. Monitor gag reflex and ability to swallow	4.51	0.826
3.13. Assess for dysphasia due to injury in area of recognition of spoken language	4.34	0.918
3.14. Feed patient safely according to poststroke severity of dysphagia	4.46	0.888
3.15. Assess need for physical restraints and apply them safely	4.51	0.817
3.16. Conduct pain assessment and analgesic administration as needed following physician’s orders	4.55	0.796
3.17. Conduct skin assessment using the Braden scale	4.52	0.826
3.18. Provide pressure relief mattress	4.55	0.787
3.19. Reposition patient every 2 h	4.50	0.826
3.20. Perform passive range-of-motion exercises to prevent contractures	4.47	0.816
3.21. Transfer patient from bed to chair or from bed to bed safely	4.48	0.854
3.22. Conduct appropriate management for patient with cognitive impairment	4.50	0.798
3.23. Neglect syndrome: teach patient to touch and use both sides of body	3.50 *	1.506
3.24. Hemianopsia: encourage patient to turn head to scan complete range of vision	3.64 *	1.468
3.25. Increase mobility as tolerated by patient and allowed by physiotherapist	4.44	0.833
3.26. Encourage independence in activities of daily living in safe manner	4.43	0.842
3.27. Refer patient to speech and language pathologist as prescribed	4.27	0.967
3.28. Screen for poststroke depression	2.76 *	1.724
3.29. Assess and report psychosocial needs	3.09 *	1.694
3.30. Encourage patient to express their feelings	3.39 *	1.567
3.31. Educate patient and family about stroke prevention and follow-up clinic after discharge	3.75 *	1.500
3.32. Refer family members to health educator to learn about how to provide home care	4.40	0.848
3.33. Provide end-of-life care for patient	4.41	0.803
3.34. Refer patient to physiatrist/rehabilitation team and assess need for rehabilitation program	4.41	0.822

* Participants disagreed or were neutral about application of the practices.

**Table 3:** Nursing practices for stroke patients during prehospital care across units.

Dimension 1: Prehospital Care		Stroke Unit N (%)	ICU N (%)	Chi-Square Tests of Independence
Practices				
Activate stroke code	Strongly disagree	4 (3.1)	37 (28.2)	X^2^ (4) = 48.34 *p* < 0.001
	Disagree	0 (0)	11 (8.4)	
	Neutral	4 (3.1)	4 (3.1)	
	Agree	18 (13.7)	8 (6.1)	
	Strongly agree	32 (24.4)	13 (9.9)	
Initiate rapid transfer to designated bed	Strongly disagree	4 (3.1)	33 (25.2)	X^2^ (4) = 48.74 *p* < 0.001
	Disagree	0 (0)	14 (10.7)	
	Neutral	2 (1.5)	4 (3.1)	
	Agree	20 (15.3)	10 (7.6)	
	Strongly agree	32 (24.4)	12 (9.2)	

ICU = intensive care unit.

**Table 4:** Nursing practices for stroke patients during acute care across units.

Dimension 2: Acute Care		Stroke Unit N (%)	ICU N (%)	Chi-Square Tests of Independence
Practices				
Assess stroke severity by applying the National Institution of Health Stroke Scale (NIHSS)	Strongly disagree	2 (1.5)	41 (31.3)	X^2^ (4) = 70.11 *p* < 0.001
	Disagree	0 (0)	12 (9.2)	
	Neutral	1 (0.8)	3 (2.3)	
	Agree	13 (9.9)	8 (6.1)	
	Strongly agree	42 (32.1)	9 (6.9)	
Evaluate the inclusion and exclusion eligibility criteria for tPA administration	Strongly disagree	2 (1.5)	7 (5.3)	X^2^ (4) = 20.14 *p* < 0.001
	Disagree	0 (0)	11 (8.4)	
	Neutral	3 (2.3)	6 (4.6)	
	Agree	12 (9.2)	23 (17.6)	
	Strongly agree	41 (31.3)	26 (19.8)	
Perform the NIHSS score 24 h post-tPA administration	Strongly disagree	1 (0.8)	35 (26.7)	X^2^ (4) = 85.90 *p* < 0.001
	Disagree	0 (0)	19 (14.5)	
	Neutral	1 (0.8)	7 (5.3)	
	Agree	12 (9.2)	6 (4.6)	
	Strongly agree	44 (33.6)	6 (4.6)	
Obtain the NIHSS score before the procedure	Strongly disagree	1 (0.8)	32 (24.4)	X^2^ (4) = 84.61 *p* < 0.001
	Disagree	0 (0)	23 (17.6)	
	Neutral	2 (1.5)	7 (5.3)	
	Agree	14 (10.7)	5 (3.8)	
	Strongly agree	41 (31.3)	6 (4.6)	
Obtain the postprocedural NIHSS score	Strongly disagree	1 (0.8)	30 (22.9)	X^2^ (4) = 78.08 *p* < 0.001
	Disagree	0 (0)	20 (15.3)	
	Neutral	0 (0)	7 (5.3)	
	Agree	17 (13)	8 (6.1)	
	Strongly agree	40 (30.5)	8 (6.1)	

NIHSS = the National Institutes of Health Stroke Scale.

**Table 5:** Nursing practices for stroke patients during postacute care across units.

Dimension 3: Postacute Care		Stroke Unit N (%)	ICU N (%)	Chi-Square Tests of Independence
Practices				
Assess patient physical disability using the modified Rankin scale or other tools	Strongly disagree	1 (0.8)	27 (20.6)	X^2^ (4) = 61.24 *p* < 0.001
	Disagree	0 (0)	22 (16.8)	
	Neutral	2 (1.5)	2 (1.5)	
	Agree	13 (9.9)	6 (6.9)	
	Strongly agree	42 (32.1)	13 (9.9)	
Neglect syndrome: teach the patient to touch and use both sides of the body	Strongly disagree	1 (0.8)	21 (16)	X^2^ (4) = 44.72 *p* < 0.001
	Disagree	0 (0)	17 (13)	
	Neutral	5 (3.8)	9 (6.9)	
	Agree	18 (13.7)	12 (9.2)	
	Strongly agree	34 (26)	14 (10.7)	
Hemianopsia: encourage the patient to turn the head to scan the complete range of vision	Strongly disagree	1 (0.8)	18 (13.7)	X^2^ (4) = 39.22 *p* < 0.001
	Disagree	0 (0)	14 (10.7)	
	Neutral	5 (3.8)	11 (8.4)	
	Agree	14 (10.7)	14 (10.7)	
	Strongly agree	38 (29)	16 (12.2)	
Screen for poststroke depression	Strongly disagree	1 (0.8)	51 (38.9)	X^2^ (4) = 101.59 *p* < 0.001
	Disagree	2 (1.5)	17 (13)	
	Neutral	6 (4.6)	1 (0.8)	
	Agree	13 (9.9)	2 (1.5)	
	Strongly agree	36 (27.5)	2 (1.5)	
Assess and report psychosocial needs	Strongly disagree	2 (1.5)	35 (26.7)	X^2^ (4) = 74.44 *p* < 0.001
	Disagree	1 (0.8)	23 (17.6)	
	Neutral	3 (2.3)	3 (2.3)	
	Agree	16 (12.2)	2 (1.5)	
	Strongly agree	36 (27.5)	10 (7.6)	
Encourage the patient to express her or his feelings	Strongly disagree	1 (0.8)	21 (16)	X^2^ (4) = 58.64 *p* < 0.001
	Disagree	1 (0.8)	27 (20.6)	
	Neutral	3 (2.3)	3 (2.3)	
	Agree	14 (10.7)	9 (6.9)	
	Strongly agree	39 (29.8)	11 (8.4)	
Educate patients and families about stroke prevention and follow-up clinic after discharge	Strongly disagree	2 (1.5)	14 (10.7)	X^2^ (4) = 40.51 *p* < 0.001
	Disagree	0 (0)	22 (16.8)	
	Neutral	3 (2.3)	2 (1.5)	
	Agree	9 (6.9)	15 (11.5)	
	Strongly agree	44 (33.6)	20 (15.3)	

## Data Availability

The data will be shared by the authors of this research paper upon request.

## Public Involvement Statement

No public involvement in any aspect of this research.

## Guidelines and Standards Statement

This manuscript was drafted against the STROBE (The Strengthening the Reporting of Observational Studies in Epidemiology) for cross-sectional studies.
